# Ambulatory Monitoring of Swallowing in Healthy Adults During Daily Activities

**DOI:** 10.3390/jcm15218692

**Published:** 2026-11-07

**Authors:** Klara A. Paszkiet, Benjamin Riebold, Bruno Fimm, Rainer O. Seidl

**Affiliations:** 1 Teaching and Research Logopedics, Clinic of Neurology, Faculty of Medicine, RWTH Aachen University, 52074 Aachen, Germany; 2 Control Systems Group, Technische Universität Berlin, 10623 Berlin, Germany; b_riebold@hotmail.com; 3 Clinic of Neurology, University Hospital RWTH Aachen, RWTH Aachen University, 52074 Aachen, Germany; bfimm@ukaachen.de; 4 Department of Otolaryngology–Head and Neck Surgery, Unfallkrankenhaus Berlin, Warener Str. 7, 12683 Berlin, Germany; rainer.seidl@charite.de

**Keywords:** swallowing, ambulatory monitoring, swallowing frequency, electromyography, bioimpedance, daily activities

## Abstract

**Background:** How often healthy adults swallow during the day and at night, and how swallowing changes around meals, remains difficult to characterize under everyday conditions. This exploratory study quantified swallowing during planned 24 h ambulatory EMG-BI monitoring and examined the practicability of prolonged recording during usual daily routines. **Methods:** Combined surface electromyography and bioimpedance recordings were obtained during participants’ usual routines. Twenty-five recordings from adults aged 19–58 years were included; 21 were complete and four were incomplete. Participants documented intake and nighttime intervals. Automated swallow detections were manually reviewed and detection errors corrected. Reviewed events were assigned to activity periods, including meals and peri-meal windows. Bioimpedance changes were used to estimate the extent and rate of pharyngeal closure. **Results:** Among 21 complete recordings, the mean total was 1041 ± 300 swallows over 24 h. Mean swallowing frequencies were 0.78 ± 0.27/min in the non-intake reference category, 3.08 ± 0.86/min during meal periods, and 5.61 ± 3.57/h during documented nighttime intervals. Frequencies were significantly higher in the five minutes before and immediately after meals than in the reference category after Holm–Bonferroni correction. Meal periods also showed greater BI-derived extent and rate of pharyngeal closure. No sex comparison remained significant. **Conclusions:** Swallowing measures varied across reported daily activity periods in this small exploratory sample. These preliminary data support further ambulatory research.

## 1. Introduction

Swallowing frequency varies between individuals and measurement settings. A review identified substantial heterogeneity in spontaneous swallowing frequency across healthy and clinical populations, highlighting the need for consistent measurement and activity definitions [1]. Brief laboratory observations may not represent daily behavior, and verbal instructions can influence swallowing [2]. Ambulatory recording offers a complementary way to examine swallowing during ordinary intake and rest.

Combined electromyography (EMG) and bioimpedance (BI) can detect swallowing-related muscle activation and impedance changes using surface electrodes [3,4,5]. Previous work supports an association between BI features and pharyngeal swallowing movements [4]. Recent studies have also investigated machine-learning-assisted EMG monitoring and multimodal wearable classification [6,7,8]. Their performance depends on the detection task, population and recording conditions.

The study aimed to determine how often healthy adults swallow over a full day and during nighttime recording, and how swallowing frequency and BI-derived pharyngeal closure measures vary across non-intake periods, meals and peri-meal periods in everyday life. It also examined the practicability of recording over 24 h using three consecutive device sections of approximately eight to nine hours each. Participants were not directly observed, and their routines were not standardized, to limit interference with habitual behavior.

## 2. Materials and Methods

### 2.1. Participants and Study Design

Adults of both sexes aged 18–60 years were recruited by convenience sampling from the investigators’ surrounding community. As the study was conducted at a university, participants were predominantly students. Health status and absence of swallowing disorders were assessed by medical history taking. Exclusion criteria were neurological or neuromuscular disease, contact allergy to adhesive materials, medication potentially affecting swallowing, acute allergy or a cold. Medication exclusions included psychotropic and neuroleptic drugs that may influence attention, salivation or neuromuscular control [9]. Male participants were asked to shave the neck before electrode application. The age range was chosen to focus the exploratory dataset on adults below the older age range in which age-related swallowing changes become increasingly relevant [10].

Before the study, a sample-size calculation using G*Power [11] informed a recruitment target of 20–40 participants, allowing for dropout and technical difficulties. Based on prior findings, large effects on swallowing frequency were anticipated for sleep and meal intake relative to spontaneous non-intake swallowing. With alpha = 0.05 and power = 0.80, the planning document reports a minimum of 12 participants for these comparisons. For pre- and post-meal comparisons, conflicting prior findings motivated two-sided testing; assuming a medium effect, alpha = 0.05 and power = 0.80, the reported required sample was 24 participants.

The study was approved by the Ethics Committee of the Faculty of Medicine at RWTH Aachen University (EK 23–208). All participants provided informed consent. Thirty participants were recruited, and 25 recordings were retained for analysis.

### 2.2. Recording System

**Figure 1:**
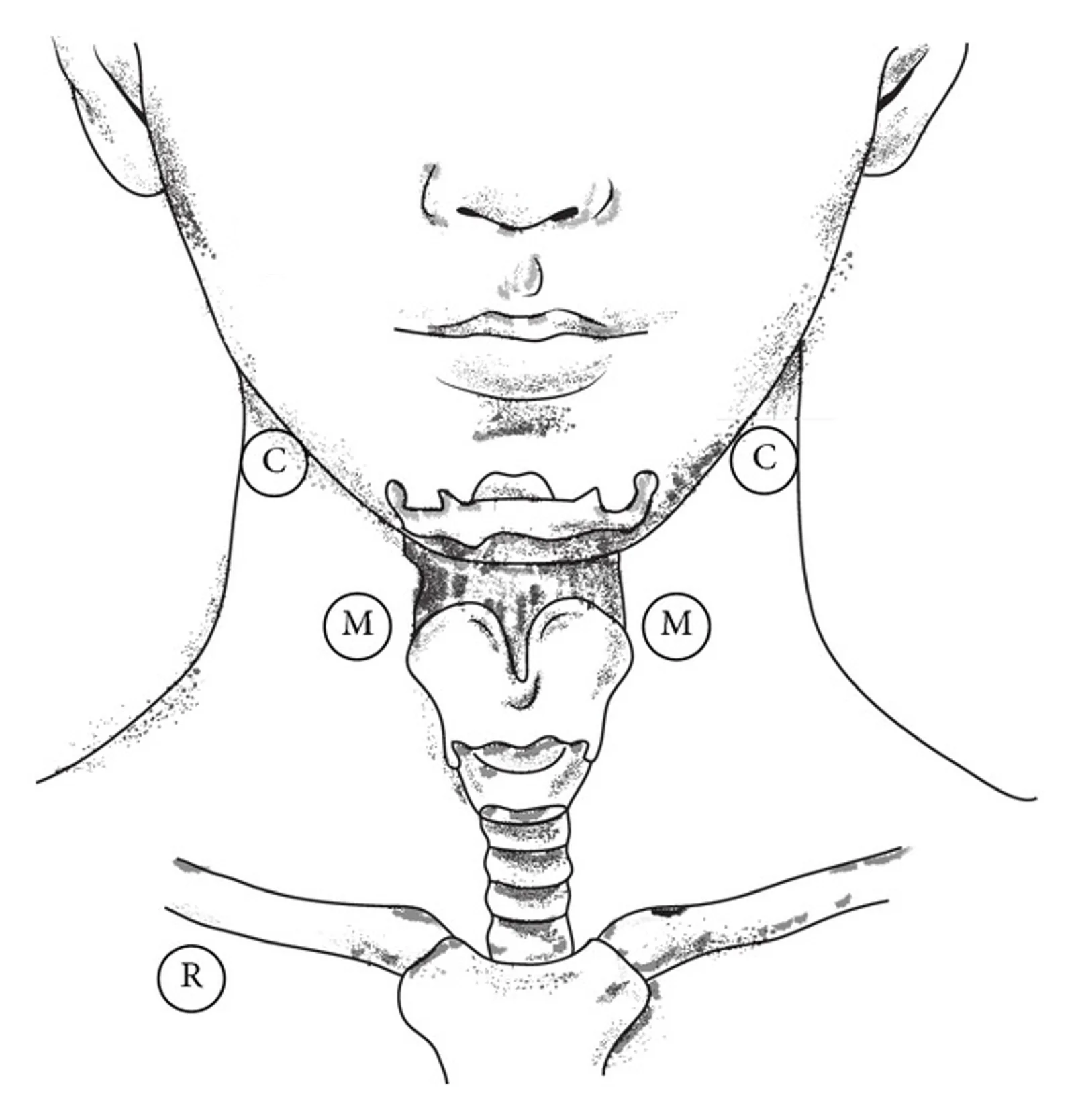
Electrode placement for combined EMG-BI recording. C denotes current-source electrodes, M voltage-measurement electrodes and R the reference electrode. Adapted from Schultheiss et al. [3].

A portable EMG-BI system (RehaIngest^®^, Hasomed, Magdeburg, Germany) recorded surface EMG and neck BI using five electrodes (Ambu^®^ BlueSensor P, Ambu A/S, Ballerup, Denmark). Two current-source electrodes were placed bilaterally at the insertion of the sternocleidomastoid muscle, two voltage-measurement electrodes between the hyoid and larynx, and a reference electrode below the clavicle [4] (Figure 1). The acquisition system used the 24-bit conversion and a sampling rate of 4000 Hz [12]. Swallowing events were detected with a random-forest procedure for finished-swallow detection [12]. Previous healthy-dataset validation reported median sensitivity of 88.8%, precision of 93.6%, F1 score of 89.7% and specificity of 99.0% [12].

### 2.3. Ambulatory Recording and Activity Documentation

Participants continued their usual daily routines during a planned 24 h observation period without direct observation by investigators. Meal timing, quantity, texture and posture were deliberately not standardized because the study sought to capture ordinary behavior with minimal procedural interference. Additional sleep-monitoring equipment was deliberately omitted to limit participant burden and potential disturbance of habitual sleep and because objective sleep assessment was outside the scope of this exploratory study. Because each device had approximately eight to nine hours of battery capacity, three devices were prepared and participants changed devices twice by reconnecting the cable. Participants received an initial introduction to the devices and their operation, along with written instructions for electrode placement, after which the electrodes were applied by a researcher. A schematic placement drawing was provided as a reference for difficulties (Figure 1). Additional adhesive tape was used to secure the electrodes and reduce detachment. Participants were asked to avoid strenuous exercise because sweat could impair electrode adhesion. Device schedules included an intended one-minute overlap, with an alarm recommended for device changes. Electrodes remained in place unless adhesion deteriorated.

Participants documented eating, drinking, sleep, toothbrushing and conversations lasting more than five minutes in a predefined diary. At the start and end of each activity, they pressed the device marker button, inserting a timestamped marker directly into the measurement record. The corresponding activity was entered in the diary, together with its start and end times; meal type and main-meal or between-meal status were also recorded. During processing, the recorded markers were assigned to the diary events. If a marker had been omitted, the documented diary time was used to identify the interval. Recording gaps and discrepancies between the measurement record and diary were clarified with the participant.

### 2.4. Processing and Activity Categories

Recordings were exported in Rich Sequence Format (RSF), pseudonymized and converted to European Data Format (EDF). Raw traces were visually inspected for artifacts. Intervals with documented technical problems, e.g., as a result of poor electrode adhesion, were excluded. When artifacts lacked corresponding diary entries, the affected interval was identified by review of the recording and excluded. The Automatic Feature Extraction (AFE) application [12] was used with the default filters: a third-order Butterworth low-pass filter with a 15 Hz cutoff was applied to BI; a third-order Butterworth high-pass filter with a 15 Hz cutoff was applied to EMG. The selected cutoff was consistent with previous processing using this measurement system [6,13].

After automated detection, an experienced investigator and co-developer of the system visually reviewed the entire recorded data stream. False-positive detections were removed, and artifacts were identified and excluded before analysis. Reviewed swallowing events were assigned to reported activity intervals in the EDF browser [14] and exported for analysis. Events occurring outside documented food or drink intake were interpreted as saliva swallows. A meal-period event denotes any detected swallow within the diary-defined meal interval, including saliva swallowing during conversation. Nighttime recording intervals denote a documented start-to-end interval and may include sleep latency or wakefulness. The analysis defined five-minute pre-meal windows relative to the first detected meal swallow, and post-meal windows relative to the last detected meal swallow. These event-based anchors differ from the diary-defined start and end of intake. The reference category excludes intervals annotated as other activities, such as intake, nighttime recording, toothbrushing, prolonged conversation and assigned peri-meal windows; it is not verified saliva-only swallowing or a standardized resting condition. Each included participant had documented at least two meals. The two longest documented meal periods per participant were selected retrospectively to represent principal meals among smaller intake periods. This selection was not predefined and did not impose a standardized meal size or composition.

**Figure 2:**
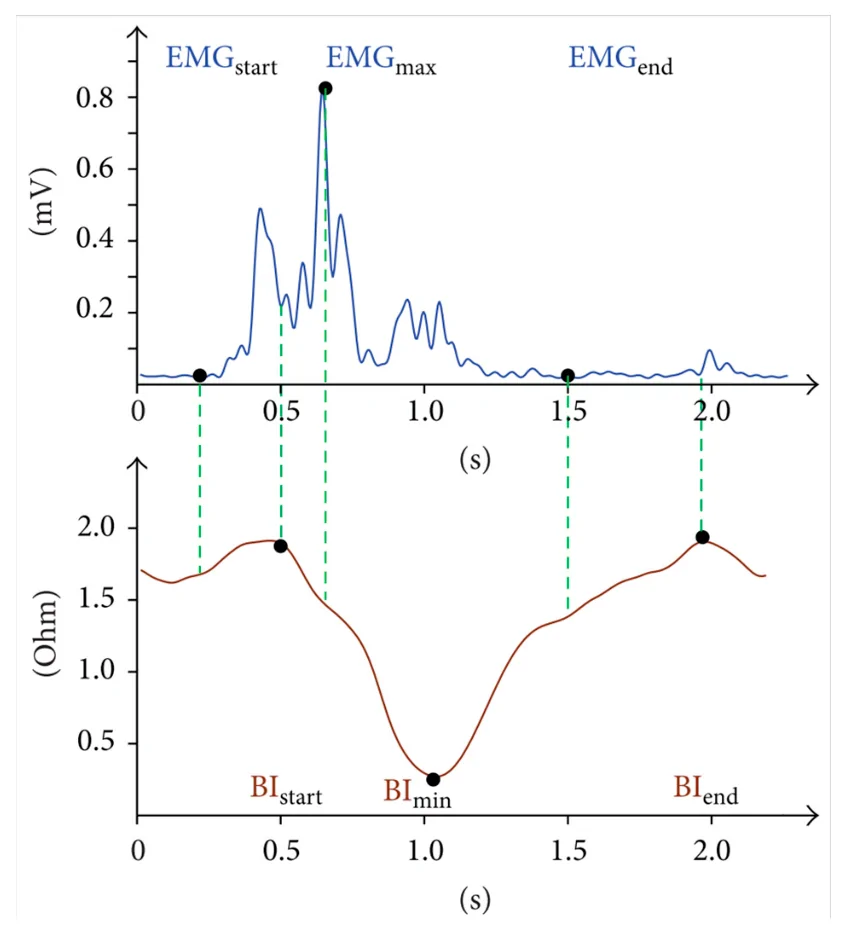
Illustrative combined EMG and BI traces of a swallow in a healthy adult. The BI decrease from swallow onset to its minimum is used to estimate the extent of pharyngeal closure; its steepest downward slope is used to estimate the rate of closure. Published example from Schultheiss et al. [3].

The analyzed outcomes were swallowing frequency, BI-derived extent of pharyngeal closure (Ω) and BI-derived rate of pharyngeal closure (Ω/s), as illustrated in Figure 2. During swallowing, closure of the normally air-filled pharyngeal space reduces the measured impedance [3,4]. The extent measure was calculated from the impedance decrease between swallow onset (BI_start) and the impedance minimum (BI_min). The rate measure was calculated from the steepest downward slope of the BI curve within that interval [3,4,12]. The tables report the positive magnitudes of these negative changes. A larger decrease represents greater BI-derived pharyngeal closure, while a steeper decrease represents faster closure. Both are relative physiological measures derived from electrical signal changes, rather than absolute anatomical dimensions or velocities in millimeters or millimeters per second.

Swallowing frequency was expressed as events per minute, with evaluation in one-minute intervals. The peri-meal categories covered the five minutes before a meal, the first five minutes after a meal and minutes five to ten after a meal. These five-minute windows define the activity categories; one minute is the resolution of frequency evaluation. Mean swallowing frequency and BI-derived extent and rate of pharyngeal closure were calculated for each participant and activity category, then summarized across participants as mean ± standard deviation.

### 2.5. Statistical Analysis

Statistical analyses were performed using SPSS Statistics 28.0. Participant summaries were reported as means ± standard deviations with 95% confidence intervals. Activity conditions were compared using paired *t*-tests, and groups stratified by sex were compared using independent-samples *t*-tests. Directional hypotheses were specified before analysis for higher meal frequency and BI measures and lower nighttime swallowing frequency and BI measures relative to the reference. These comparisons used one-sided tests; peri-meal change hypotheses and further exploratory tests were two-sided. Holm–Bonferroni correction was applied separately to two families, each containing 18 comparisons: activity comparisons and sex comparisons. Raw *p* values are identified in the tables; significance markings refer to the corrected decisions at family-wise alpha = 0.05.

## 3. Results

### 3.1. Participants and Available Recordings

**Figure 3:**
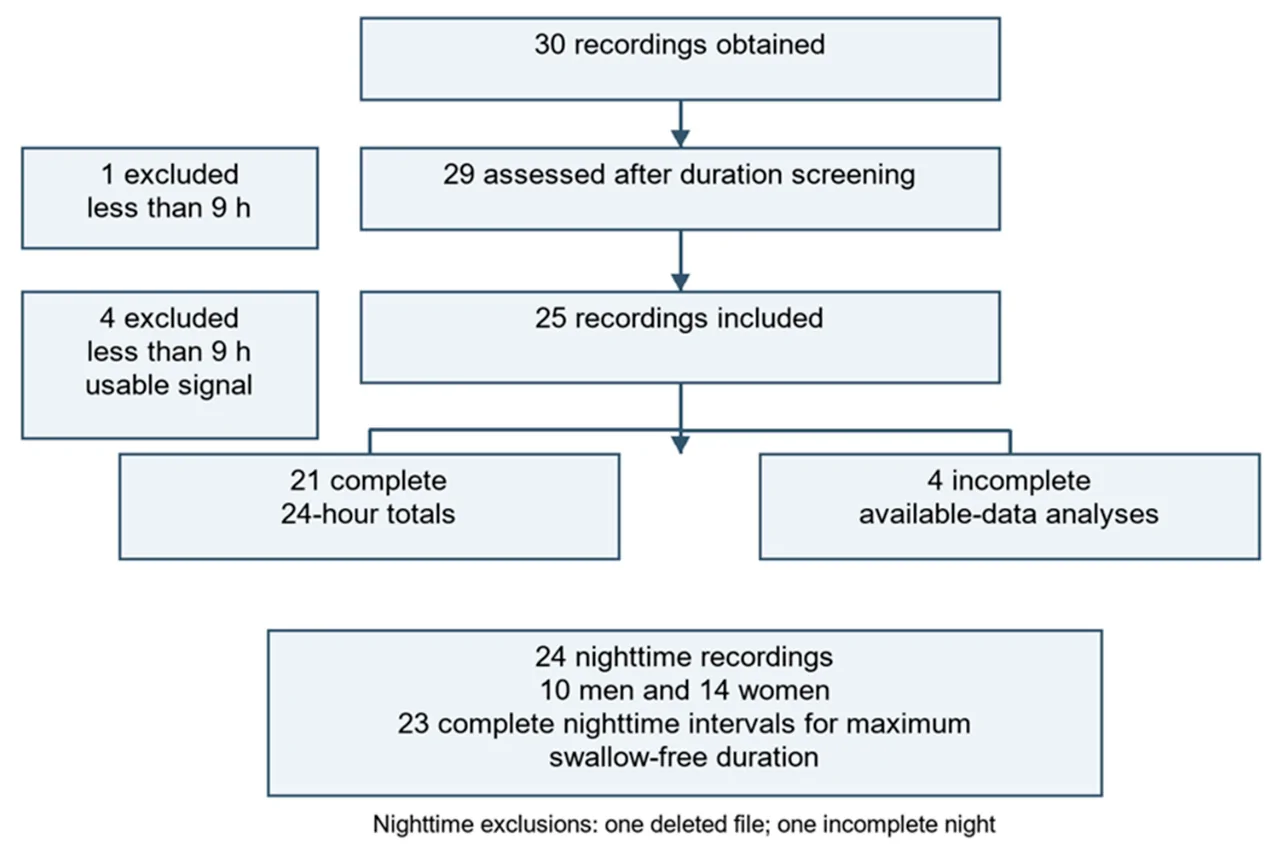
Participant and recording flow. Incomplete recordings contributed only to endpoints with available data; complete nighttime coverage was required for the maximum swallow-free interval.

**Figure 4:**
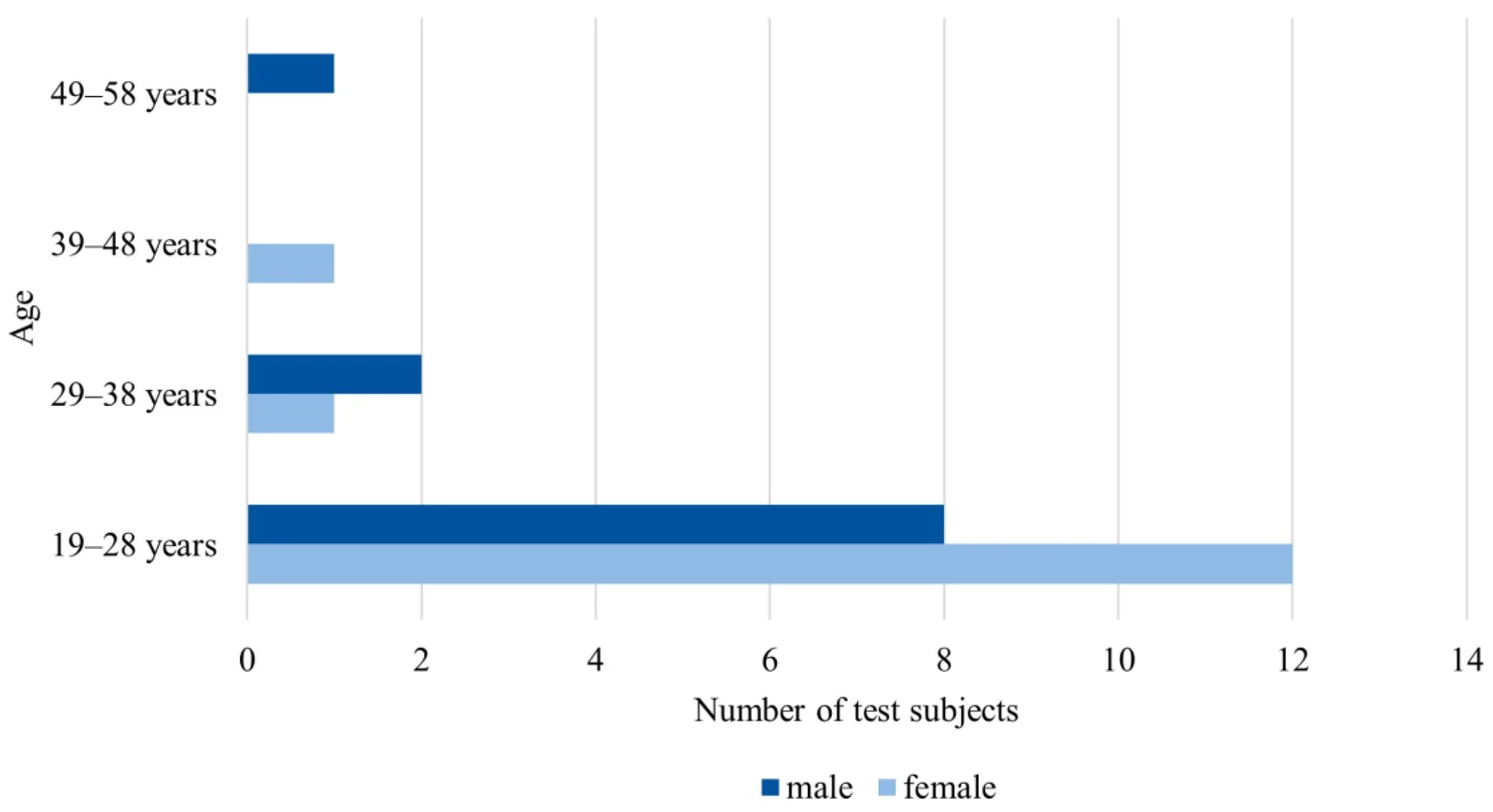
Age distribution of the 25 included participants by sex. Twenty participants were aged 19–28 years.

Recording took place from January to May 2024. Of 30 recordings, one was excluded for recording duration below nine hours and four further recordings because signal interference left fewer than nine hours of usable data. In two men, hair-related interference impaired signal quality; in two women, insufficient electrode adhesion was documented. The >9 h inclusion threshold was motivated by the approximately nine-hour battery duration of a single recorder; it was a technical rule rather than a validated minimum for whole-day representativeness. Twenty-five recordings were included: 21 complete and four incomplete. Each retained incomplete recording still contained more than nine hours of measurement data and contributed to analyses for which usable data were available (Figure 3). The included participants were 14 women and 11 men aged 19–58 years. Twenty of the 25 participants were aged 19–28 years (Figure 4).

Among 21 complete recordings, the mean total was 1041 ± 300 detected swallowing events over 24 h (range 638–1789). The four incomplete recordings contributed only to analyses with available data and were not extrapolated to estimate whole-day totals.

### 3.2. Activity-Associated Patterns

Swallowing frequency was 0.78 ± 0.27 events/min in the non-intake reference category. It was significantly higher during meals (3.08 ± 0.86/min), in the five minutes before meals (1.03 ± 0.42/min), immediately after meals (1.19 ± 0.52/min), and significantly lower during nighttime recording (5.61 ± 3.57/h); all corresponding raw *p* values were <0.001 and remained significant after the Holm–Bonferroni correction (Table 1 and Table 2). Five to ten minutes after meals, frequency was 0.89 ± 0.42/min and did not differ significantly from the reference (raw *p* = 0.115). The BI-derived extent of pharyngeal closure was significantly greater during meals (1.48 ± 0.28 Ω) than in the reference category (1.14 ± 0.24 Ω; raw *p* < 0.001); the other displayed extent comparisons were nonsignificant after correction. The BI-derived rate of pharyngeal closure was significantly greater during meals (3.20 ± 0.74 Ω/s; raw *p* < 0.001), immediately after meals (2.34 ± 0.67 Ω/s; raw *p* = 0.003), and lower during nighttime recording (1.71 ± 0.49 Ω/s; raw *p* < 0.001), compared with the reference (2.11 ± 0.53 Ω/s). The pre-meal and five-to-ten-minute post-meal rates were 2.17 ± 0.57 and 2.18 ± 0.66 Ω/s, respectively, without significant differences from the reference.

Nighttime measures were available for 24 participants (10 men and 14 women). In one incomplete recording, a male participant accidentally deleted the files from the recorder used during the night, leaving no nocturnal signal; this recording was excluded from nighttime analyses. In another incomplete recording, most nocturnal data were available, but electrodes detached near the end of the night. Its available data contributed to swallowing frequency and BI measures, but the recording was excluded from the longest swallow-free interval analysis because the end of the documented nighttime interval was not covered. Accordingly, the nighttime swallowing rate was 5.61 ± 3.57 events/h in 24 participants, whereas the mean longest event-free interval was 79.1 ± 34.5 min in 23 participants with complete nighttime recording coverage (range 31.9–177.6 min).

**Table 2:** Activity comparisons with non-intake swallowing. (**a**) Differences in swallowing frequency relative to non-intake swallowing. (**b**) Differences in BI-derived extent of pharyngeal closure relative to non-intake swallowing. (**c**) Differences in BI-derived rate of pharyngeal closure relative to non-intake swallowing.

(**a**)				
Outcome and Comparison	*n*	*p* Raw	Holm Significant *	d z (approx.)
Pre-meal 5 min	25	<0.001	*	0.81
Meal period	25	<0.001	*	2.85
Post-meal 0–5 min	25	<0.001	*	1.03
Post-meal 5–10 min	25	0.115	No	0.33
Nighttime recording interval	24	<0.001	*	−2.48
(**b**)				
Outcome and comparison	*n*	*p* raw	Holm significant *	d z (approx.)
Pre-meal 5 min	25	0.033	No	0.45
Meal period	25	<0.001	*	1.64
Post-meal 0–5 min	25	0.187	No	0.27
Post-meal 5–10 min	25	0.530	No	0.13
Nighttime recording interval	24	0.009	No	−0.52
(**c**)				
Outcome and comparison	*n*	*p* raw	Holm significant *	d z (approx.)
Pre-meal 5 min	25	0.387	No	0.18
Meal period	25	<0.001	*	1.88
Post-meal 0–5 min	25	0.003	*	0.67
Post-meal 5–10 min	25	0.301	No	0.21
Nighttime recording interval	24	<0.001	*	−1.07

*p* values are unadjusted. Meal and nighttime comparisons with the non-intake reference used one-sided paired tests; other displayed comparisons used two-sided paired tests. * denotes significance after the Holm–Bonferroni correction within the single 18-comparison activity family (family-wise alpha = 0.05). Significance decisions are retained from the original corrected analysis. d_z is an approximate standardized paired difference calculated as *t*/√*n* from rounded *t* statistics and participant numbers; negative values indicate lower values than the reference.

### 3.3. Sex Comparisons

The included sample comprised 14 women and 11 men. Table 3 reports raw *p* values. None of its 18 comparisons betweeen sexes remained significant after the separate Holm–Bonferroni correction; nominal raw *p* values below 0.05 must not be interpreted as significant sex differences.

**Table 3:** Sex differences in swallowing frequency and BI-derived pharyngeal closure measures. (**a**) Swallowing frequency in women and men in events per minute. (**b**) BI-derived extent of pharyngeal closure in women and men in ohms. (**c**) BI-derived rate of pharyngeal closure in women and men in ohms per second.

(**a**)					
Activity	Men M ± SD	Women M ± SD	*p* Raw	*p* Holm †	g †
Non-intake reference	0.66 ± 0.24	0.88 ± 0.26	0.042	0.588	0.85
Pre-meal 5 min	0.92 ± 0.47	1.11 ± 0.36	0.250	1.000	0.45
Meal period	2.99 ± 0.93	3.15 ± 0.83	0.635	1.000	0.18
Post-meal 0–5 min	0.97 ± 0.41	1.36 ± 0.54	0.063	0.756	0.77
Post-meal 5–10 min	0.76 ± 0.32	0.99 ± 0.48	0.197	1.000	0.53
Nighttime recording interval	0.09 ± 0.08	0.09 ± 0.04	0.914	1.000	0.00
(**b**)					
Activity	Men M ± SD	Women M ± SD	*p* raw	*p* Holm †	g †
Non-intake reference	1.07 ± 0.26	1.20 ± 0.23	0.209	1.000	0.52
Pre-meal 5 min	1.14 ± 0.29	1.24 ± 0.27	0.365	1.000	0.35
Meal period	1.43 ± 0.31	1.53 ± 0.26	0.384	1.000	0.34
Post-meal 0–5 min	1.05 ± 0.29	1.29 ± 0.24	0.032	0.480	0.88
Post-meal 5–10 min	1.03 ± 0.35	1.27 ± 0.28	0.073	0.803	0.74
Nighttime recording interval	0.96 ± 0.22	1.06 ± 0.23	0.312	1.000	0.43
(**c**)					
Activity	Men M ± SD	Women M ± SD	*p* raw	*p* Holm †	g †
Non-intake reference	1.88 ± 0.42	2.30 ± 0.54	0.049	0.637	0.83
Pre-meal 5 min	1.88 ± 0.42	2.39 ± 0.58	0.020	0.340	0.95
Meal period	2.93 ± 0.78	3.42 ± 0.65	0.099	0.990	0.67
Post-meal 0–5 min	1.96 ± 0.50	2.64 ± 0.64	0.008	0.144	1.13
Post-meal 5–10 min	1.85 ± 0.40	2.44 ± 0.72	0.023	0.368	0.95
Nighttime recording interval	1.53 ± 0.39	1.84 ± 0.52	0.125	1.000	0.64

† Holm adjustment reconstructed within the authors-confirmed sex-comparison family of 18 tests; numerical values use rounded source *p* values. All 18 corrected decisions are nonsignificant. Hedges g is an approximate descriptive reconstruction from rounded means/SDs using *n* men = 11 and *n* women = 14 for non-nighttime rows, and *n* men = 10 and *n* women = 14 for nighttime recording. Pooled SD and the small-sample correction 1 − 3/(4df − 1) are used, with df = *n* men + *n* women − 2. Positive values indicate larger means in women. Rounded nighttime frequency means can yield g = 0.00 despite a nonzero original *t* statistic; this is a rounding limitation. Table 3 uses 11 men and 14 women in the five non-nighttime conditions and 10 men and 14 women in the nighttime condition. The original subgroup CIs are omitted here to reduce duplication; confidence intervals for sex differences were not reconstructed from rounded descriptive values.

## 4. Discussion

This exploratory study quantified everyday swallowing across a full day and nighttime recording and examined activity-associated swallowing patterns. Complete 24 h recordings yielded 1041 ± 300 swallows per person, while nighttime frequency was 5.61 ± 3.57 events/h. The 0.78/min non-intake reference rate describes a specific activity category and is not the whole-day rate. The consecutive-device protocol also demonstrated the practicability and practical limits of ambulatory acquisition. 21 of the 30 recordings were complete, while four additional incomplete recordings were retained for available-data analyses. These counts indicate that prolonged ambulatory recording was achievable for many participants, but also identify signal quality and incomplete coverage as practical constraints. Meal periods showed higher event rates, larger BI-derived closure extents and higher BI-derived closure rates than the non-intake reference category. Nighttime recording intervals showed lower event rates and lower BI-derived closure rates. The reference rate of 0.78/min is within the range summarized for healthy younger groups in previous research [1], although activity definitions and observation conditions limit direct comparison.

The meal category includes all detected events within the reported interval, including saliva swallowing during conversation. Consequently, meal-period frequency should not be interpreted as a rate of food-bolus swallows alone. Food texture, bolus volume, meal composition, hydration, preparation, conversation and posture were deliberately not standardized to preserve usual daily behavior. This choice is appropriate for the exploratory naturalistic aim, but limits causal interpretation and controlled comparisons. Meal-associated BI differences cannot therefore be attributed to a particular bolus property or anatomical movement.

Swallowing frequency increased in the periods immediately before and after meals. This describes when the increase occurred, but does not establish what caused it. Responses to food cues and changes in salivation are possible explanations [15,16]; salivary flow and cortical activity were not measured. Drinking, conversation, movement or postural changes around meals may also contribute. The two longest meals may differ systematically from shorter meals, limiting interpretation of the selected meal periods.

During documented nighttime intervals, participants swallowed 5.61 ± 3.57 times per hour. This was descriptively higher than the 2.4 ± 1.0 events/h reported by Sato et al. during sleep [17]. The mean longest swallow-free interval was 79.1 ± 34.5 min, compared with 68.8 ± 24.8 min in that study [17]. Our nighttime intervals were defined by participant reports and may include sleep latency and intermittent wakefulness. Differences in interval definition and measurement conditions may therefore contribute to the observed values. No objective sleep staging was performed, so the results cannot establish an association with sleep depth.

BI measurements reflect relative electrical changes during pharyngeal closure. The reduction in the air-filled pharyngeal space during swallowing lowers impedance; the amplitude and steepest slope of this decrease provide estimates of closure extent and rate [3,4]. BI does not provide an absolute anatomical distance or movement velocity. The resulting values depend on factors such as body posture and swallowing conditions [18] and cannot be converted into universal millimeter reference values. The present healthy sample provides preliminary distributions for this measurement method rather than diagnostic cutoffs for impaired pharyngeal closure.

Each participant underwent one ambulatory recording. The study therefore describes variation within the recorded day and between participants, but does not determine whether the same person would produce comparable results on another day. Repeated ambulatory measurements are needed to assess this day-to-day reproducibility.

Clinical translation requires studies in older adults and patients with dysphagia, including comparison with appropriate clinical or instrumental reference assessments, evaluation of performance under ordinary activities, and external validation. Clinical studies of spontaneous swallowing frequency [19] provide motivation, but the current healthy sample cannot establish screening thresholds, aspiration risk or therapeutic benefit. No corrected comparison betweeen sexes demonstrated a difference, and small subgroups preclude firm conclusions.

### Limitations

The convenience sample was small, predominantly young and largely recruited among students in a university setting, restricting generalizability and precision, particularly for comparisons betweeen sexes. Health and absence of swallowing disorders were assessed anamnestically rather than established by an instrumental reference examination. Four retained recordings were incomplete. Missing intervals may be associated with activity, posture or electrode adhesion and therefore may not be random; available-time rates do not necessarily recover whole-day behavior. Participant-reported timing introduces classification uncertainty for meals and sleep, and interval categories do not directly establish bolus origin. Daily intake, hydration and posture were uncontrolled; strenuous exercise was discouraged to preserve electrode adhesion. Incomplete recordings contributed only to analyses with usable data. The missing signal was not counted as swallow-free time when determining the longest nighttime interval.

The automated detections were manually checked and corrected throughout the recorded data stream. An independent reference assessment of every swallowing event would be needed to quantify detection accuracy under these everyday conditions. Such continuous reference monitoring was not available for this ambulatory protocol, so the present study cannot provide independently verified sensitivity or specificity for daily life recording.

Device markers recorded activity changes directly in the signal and were linked to the diary entries. Timing uncertainty arose primarily when participants omitted or delayed a marker and the activity interval had to be reconstructed from the diary. Artifact-contaminated intervals were excluded after visual review, but corrections were not systematically counted; their frequency therefore cannot be quantified.

Wearing the device, changing recorders and documenting activities may themselves alter behavior; omission of direct observation and additional equipment reduces procedural interference but does not prove absence of reactivity.

Extended observation can improve sampling of one person’s behavior, but does not remove biological interindividual variability, systematic detection error or selection bias. These findings are preliminary distributions rather than population reference limits.

## 5. Conclusions

Healthy adults in this exploratory sample swallowed an average of 1041 ± 300 times over complete 24 h recordings. During documented nighttime intervals, the mean rate was 5.61 ± 3.57 events/h, and the mean longest swallow-free interval was 79.1 ± 34.5 min. Compared with the non-intake reference (0.78 ± 0.27/min), swallowing frequency was higher during meals (3.08 ± 0.86/min), in the five minutes before meals (1.03 ± 0.42/min) and immediately after meals (1.19 ± 0.52/min). Meal periods also showed greater BI-derived extent and rate of pharyngeal closure. These findings characterize everyday swallowing across day and night and demonstrate the practicability of prolonged ambulatory recording with consecutive devices. Repeated measurements and studies in clinical populations are needed to assess reproducibility and clinical applicability.

## Figures and Tables

**Table 1:** Swallowing frequency and BI-derived pharyngeal closure measures by daily activity. (**a**) Swallowing frequency by activity in events per minute. (**b**) BI-derived extent of pharyngeal closure by activity in ohms. (**c**) BI-derived rate of pharyngeal closure by activity in ohms per second.

(**a**)			
Activity	*n*	Mean ± SD	95% CI
Non-intake reference	25	0.78 ± 0.27	0.67–0.89
Pre-meal 5 min	25	1.03 ± 0.42	0.86–1.20
Meal period	25	3.08 ± 0.86	2.73–3.44
Post-meal 0–5 min	25	1.19 ± 0.52	0.97–1.40
Post-meal 5–10 min	25	0.89 ± 0.42	0.71–1.06
Nighttime recording interval	24	0.09 ± 0.06	0.07–0.12
(**b**)			
Activity	*n*	Mean ± SD	95% CI
Non-intake reference	25	1.14 ± 0.24	1.04–1.24
Pre-meal 5 min	25	1.20 ± 0.28	1.08–1.31
Meal period	25	1.48 ± 0.28	1.37–1.60
Post-meal 0–5 min	25	1.18 ± 0.29	1.06–1.30
Post-meal 5–10 min	25	1.16 ± 0.32	1.03–1.30
Nighttime recording interval	24	1.02 ± 0.23	0.92–1.11
(**c**)			
Activity	*n*	Mean ± SD	95% CI
Non-intake reference	25	2.11 ± 0.53	1.90–2.33
Pre-meal 5 min	25	2.17 ± 0.57	1.93–2.40
Meal period	25	3.20 ± 0.74	2.90–3.51
Post-meal 0–5 min	25	2.34 ± 0.67	2.06–2.61
Post-meal 5–10 min	25	2.18 ± 0.66	1.91–2.46
Nighttime recording interval	24	1.71 ± 0.49	1.50–1.92

*n* = 25 for non-nighttime categories and *n* = 24 for nighttime recording. Values are participant-level means ± SD and 95% confidence intervals. Nighttime frequency is rounded to 0.09/min; the directly reported value is 5.61/h. The non-intake reference rate is not the frequency averaged over all 24 h.

## Data Availability

The data presented in this study are available from the corresponding author on reasonable request. The data are not publicly available due to restrictions imposed by the participants’ consent and the approved study protocol.

## Abbreviations

The following abbreviations are used in this manuscript:

EMG	Electromyography
BI	Bioimpedance
RSF	Rich Sequence Format
EDF	European Data Format
SD	Standard deviation
CI	Confidence interval
